# Reliability of the Swedish version of the Evidence-Based Practice Attitude Scale assessing physiotherapist’s attitudes to implementation of evidence-based practice

**DOI:** 10.1371/journal.pone.0225467

**Published:** 2019-11-25

**Authors:** Kirsti Skavberg Roaldsen, Alexandra Halvarsson

**Affiliations:** 1 Division of Physiotherapy, Department of Neurobiology, Care Sciences and Society, Karolinska Institutet, Huddinge, Sweden; 2 Department of Research, Sunnaas Rehabilitation Hospital, Nesoddtangen, Norway; 3 Department of Physiotherapy, Faculty of Health Sciences, Oslo Metropolitan University, Oslo, Norway; 4 Allied Health Professionals Function, Karolinska University Hospital, Stockholm, Sweden; Medical University Innsbruck, AUSTRIA

## Abstract

**Purpose:**

To translate and apply a cross-cultural adaptation of the Evidence-Based Practice Attitude Scale (EBPAS) in Swedish and investigate its absolute and relative reliability.

**Methods:**

The original EBPAS (a questionnaire assessing health professionals’ attitudes to implementation of evidence-based practice) was translated into Swedish using a forward and backward procedure, including a group discussion and expert committee. To assess reliability, 55 physiotherapists (48 women) aged 23–64 years from different clinical settings in the Stockholm region answered the EBPAS by postal survey twice within an interval of 2 weeks.

**Results:**

The Cronbach’s alpha values for EBPAS were >0.721. The intraclass correlation (ICC) between test and retest (relative reliability) was moderate to good for the four subscales, with ICC(A.1) and ICC(C.1) values approximately equal and in the range 0.56–0.89. Values for the absolute reliability of the mean score were a standard error of measurement of about 7% and a smallest real difference of about 19%.

**Conclusion:**

The Swedish version of the EBPAS shows mainly good reliability.

## Introduction

Evaluation of the transition process from evidence-based intervention methods into clinical practice in implementation research relies on tools that measure outcomes, such as acceptability, feasibility, fidelity and sustainability. There are many research studies reporting on the efficacy and effectiveness of certain treatments, some of which may have taken many years for the researchers to develop and evaluate in controlled research settings.

Moreover, many researchers do not translate their findings into routine practice by taking their results into everyday clinical practice and if they do it often take considerable time. This delay means that possible health gains are not achieved as quickly as one would have hoped for based on the potential patient benefits of the research findings. This gap between what is known and what is consistently done in health care, often referred to as the knowing-doing gap [1]. Therefore, it is important that treatments and methods proven to have beneficial effects for patients are implemented in clinical practice. It is also important to evaluate different strategies to achieve successful implementation to determine which strategies are most effective in reducing the knowing-doing gap, i.e. to perform implementation research.

The Evidence-Based Practice Attitude Scale (EBPAS) can be used to assess acceptability in implementation research [2]. However, this questionnaire is not available in Swedish; in research, translated, valid and reliable assessments are required. Therefore, the aim of this study was to translate and apply a cross-cultural adaptation of the EBPAS to Swedish and to investigate its absolute reliability (measurement error) as well as relative reliability (intraclass correlation) within a Swedish-speaking sample of physiotherapists.

## Methods

Before the study was conducted, the first author contacted the original author of EBPAS, Professor Gregory A. Aarons, United States, and he gave his approval for a Swedish translation. The ethical application was approved in 2016 by the Regional Ethical Board in Stockholm, Sweden (Dnr. 2016/415-31) and all participants gave written informed consent to participate.

### Evidence-Based Practice Attitude Scale

The EBPAS [2] consists of 15 items and is designed to measure general attitudes to implementation of evidence-based practice. Each item is answered on a five-point scale from 0 (not at all) to 4 (agree completely). The questionnaire can be divided into four different scales (scale 1–4): scale 1 (requirements) refers to whether practitioners will use the innovation if it is requested by the service, supervisor or by agency mandates and consists of questions 11, 12 and 13; scale 2 (appeal) refers to whether practitioners will use an innovation if it is attractive, gives meaning, can be used correctly, or is being used by colleagues who are pleased with it and consists of questions 9, 10, 14 and 15; scale 3 (openness) is the degree to which practitioners are willing to try new interventions and consists of questions 1, 2, 4 and 8; scale 4 (divergence) refers to whether practitioners experience research-based interventions as not clinically useful and less important than clinical experience and consists of questions 3, 5, 6 and 7.

According to Aarons [2], scoring of each subscale is created by computing a mean score for each set and higher scores indicate more favourable attitudes. For scale 4 (divergence), each item must be reversed before computing the subscale mean.

### Translation and cross-cultural adaptation

**Fig 1:**
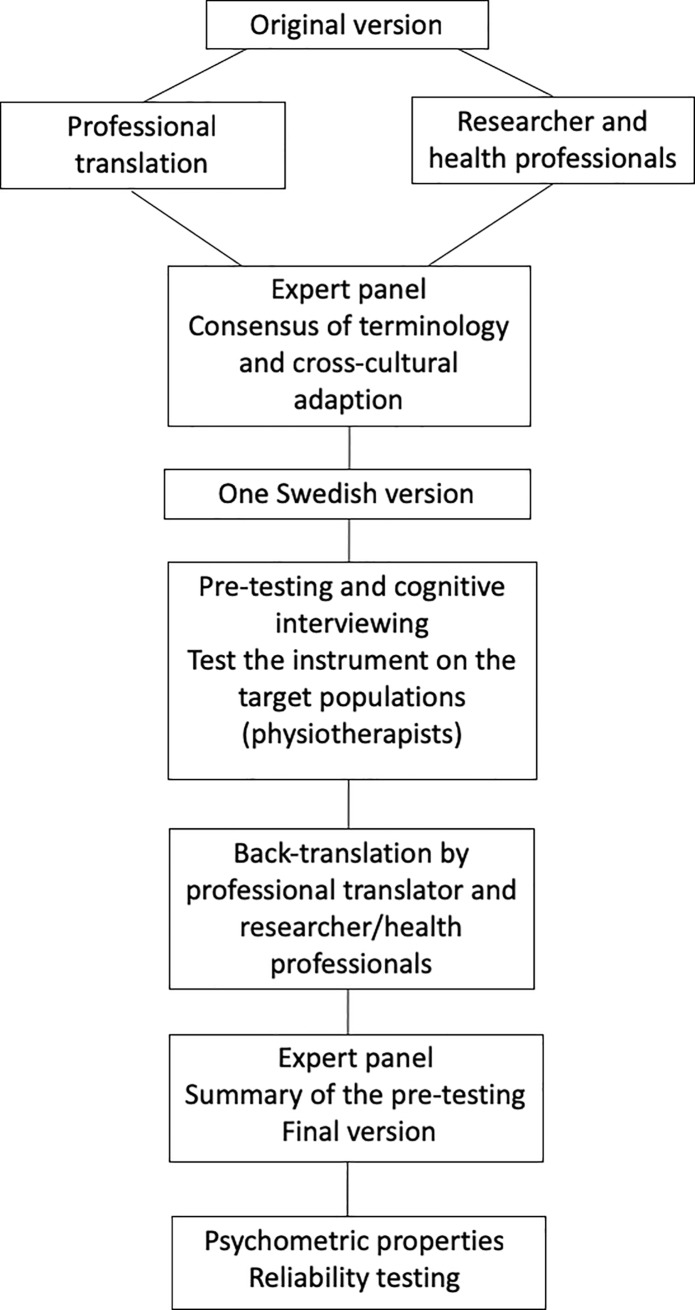
The translation and reliability procedure for the Swedish version of the Evidence-Based Practice Attitude Scale.

The EBPAS was translated and back-translated according to guidelines for cross-cultural adaptation of self-reporting instruments [3] (Fig 1). Initially, one professional translator and two bilingual physiotherapists familiar with the terminology translated the EBPAS from English to Swedish separately. The translations emphasized conceptual and cultural interpretations rather than literal translations. Thereafter, an expert panel comprising four physiotherapists with clinical and/or research expertise in the area of physiotherapy and evidence-based practice met to compare the versions. The recommendations were summarized and inadequate expressions and concepts in the translation were identified and resolved before consensus on the first version was reached.

To reach conceptual equivalence, a group discussion consisting of clinical working physiotherapists representing the target group, led by an experienced interviewer, took place to gather comments on the first version. The clinician’s representatives included five women with varied experience of evidence-based practice. The discussion was taped and later transcribed. The demographic data of the participants in this group discussion are shown in Table 1.

**Table 1:** Characteristics of the study sample.

	Group discussion translation procedure (n = 5)	Reliability testing (n = 55)
Age (years), median (min–max)	42 (27–63)	39 (23–64)
Gender (%), female/male	100/0	87/13
Professional experience (years), median (min–max)	18 (2–28)	10 (1–37)
Academic degree, Bachelor/Master/PhD (%)	60/40/0	62/35/3
Specialized certificate (%)	40	25.5

A Swedish-English back-translation of the first version were performed by three independent translators (one professional translator and two health professionals). As in the initial translation, the emphasis in the back-translation was on conceptual and cultural equivalence and not linguistic equivalence.

The expert panel then met for the second time to discuss the back-translation and issues from the group discussion, i.e. difficulty in interpreting the translated response options, the concept of manualized therapy/interventions (item 2, 6,7), the term academic researchers (item; 3) and items included in subscale 1 (Requirements) were discussed.

Finally, different interpretations, modifications and discrepancies were discussed by the expert panel of eight health professionals to reach a cross-cultural adaptation and a satisfactory final version. All the physiotherapists involved had profound knowledge of Swedish language and culture, had long clinical and/or research experience in evidence-based practice and were familiar with the instrument. See S1 Appendix for the final version of the Swedish version of EBPAS.

### Reliability

To assess absolute and relative reliability of the final Swedish version of the EBPAS, 90 physiotherapists from different clinical settings in Stockholm county were asked to participate, i.e. convenience sampling. The EBPAS was administered by postal survey to those 66 physiotherapists who agreed to participate; 56 physiotherapists answered both postal surveys. For more detailed information about the participants, see Table 1.

All participants gave written informed consent to participate and the study was approved by the Local Ethics committee in Stockholm, Sweden (Dnr. 2016/415-31).

To determine test–retest reliability, the participants answered the EBPAS a second time, also by a postal survey, 2 weeks after the first survey. To control for bias during the period between the two postal surveys, the participants answered a complementary question on whether anything had happened that could influence the outcome of the study since they had answered the first survey. Missing items in the EBPAS were replaced by calculating the sum score of the items that had been completed divided by the number of completed items. Two questionnaires from the first postal survey had missing data (1 and 2 items) and one questionnaire from the second postal survey had missing data (1 item). One participant was excluded from the analysis because of missing data in half of the items in the first postal survey. Thus, 55 physiotherapists were included in the study (age, 23–64 years; median, 39 years); 48 women and 7 men, working in different clinical settings in Stockholm county, i.e. university hospital (n = 21), hospital (n = 2), primary care (n = 24), private practice (n = 2), residential care (n = 1) and specialized rehabilitation practice (n = 5). For more detailed information about the participants, see Table 1.

### Statistical analysis

All statistical analysis was performed using IBM SPSS, version 24.0 (SPSS Inc., Chicago, Illinois, USA). Descriptive data are presented as the median, min–max, number (n) and percentage (%). Internal consistency reliability was evaluated using Cronbach’s alpha for the mean score of the total score and each subscale score. A value >0.7 indicates acceptable internal consistency reliability [4].

To establish the relative reliability, the intraclass correlation coefficients ICC(A,1) (absolute agreement) and ICC(C,1) (consistency) [5] were calculated as

ICC(A,1)=(MSBS−MSE)/(MSBS+(k−1)MSE+k/n(MSBM−MSE))

ICC(C,1)=(MSBS−MSE)/(MSBS+(k−1)MSE)

where MSBS is the mean square between subjects, MSBM is the mean square between measurements and MSE is the mean square error, all calculated by repeated measures ANOVA, including 95% confidence intervals. An ICC(A,1) value substantially lower than ICC(C,1) indicates a bias (systematic difference) between test and retest. If both ICC values are about the same, bias may be negligible. An F test using F = MSBM/MSE may be used to confirm whether non-negligible biases are present. [6]ICC values were classified qualitatively according to Bland and Altman [7]: <0.20, poor; 0.21–0.40, fair; 0.41–0.60, moderate; 0.61–0.80, good; 0.81–1.00, very good.

To quantify measurement error, we calculated absolute reliability, i.e. the standard error of measurement (SEM) and the relative SEM (SEM as a percentage of the mean value), as well as the smallest real difference (SRD) and relative SRD (SRD%) The SRD is the smallest clinically relevant change on an individual level and the SEM is the measurement error.[8, 9]. SEM was calculated as √MSWS and the SRD was calculated as SEM × √2 × 1.96. In addition, we used Bland and Altman analyses to visually assess systematic changes of the mean [8].

Sample size for the reliability testing was calculated using the 2c^2^ method [10], where c is the number of categories/response options in the questionnaire (i.e. 2 × 5^2^), resulting in a minimum of 50 participants.

## Results

A total of 55 participants were included in this analysis, i.e. answered both postal surveys with EBPAS. The results of the reliability analyses are shown in Tables 2, 3 and 4. Internal consistency was sufficient, with Cronbach’s alpha >0.83 for the total score and three of the subscales; subscale 2 (appeal) had an alpha of 0.721.

**Table 2:** Number of participants, mean, standard deviation (SD) and min–max for the total score and mean scores for the Evidence-Based Practice Attitude Scale (n = 55).

	Test 1			Test 2		
	Mean	SD	Min–max	Mean	SD	Min–max
Total	3.05	0.35	2.47–3.80	2.99	0.41	1.67–3.73
Requirements	3.15	0.77	1–4	3.10	0.79	1.67–4
Appeal	3.31	0.50	2–4	3.20	0.65	1–4
Openness	3.07	0.52	1.75–4	3.00	0.63	1–4
Divergence	1.35	0.67	0–2.75	1.32	0.60	0–2.5

**Table 3:** Cronbach’s alpha and intraclass correlation (ICC(A.1) and ICC(C.1)) with 95% confidence interval (95% CI) for the Evidence-Based Practice Attitude Scale.

	Cronbach’s alpha	ICC(A.1)	95% CI	ICC(C.1)	95% CI	*f* value	*p* value
Total	0.831	0.707	0.547–0.818	0.829	0.707–0.900	2.062	0.157
Requirements	0.890	0.803	0.685–0.880	0.891	0.813–0.936	0.407	0.526
Appeal	0.721	0.559	0.349–0.716	0.717	0.518–0.834	2.266	0.138
Openness	0.827	0.705	0.543–0.816	0.827	0.704–0.899	1.129	0.293
Divergence	0.847	0.738	0.589–0.838	0.849	0.741–0.912	0.258	0.613

**Table 4:** Standard error of measure (SEM), variation of measurement error (SEM%) and smallest real difference (SRD), as well as the mean difference between the two test sessions (*d*), 95% confidence interval (95% CI) of *d* and limits of agreement (LOA) for the Evidence-Based Practice Attitude Scale.

	SEM	SEM%	SRD	SRD%	*d*	95% CI	LOA
Total	0.205	6.79	0.568	18.81	−0.056	−0.134–0.022	−0.632–0.520
Requirements	0.346	11.10	0.960	30.77	−0.042	−0.176–0.091	−1.028–0.944
Appeal	0.385	11.80	1.066	32.71	−0.109	−0.254–0.036	−1.183–0.965
Openness	0.315	10.36	0.872	28.72	−0.064	−0.184–0.056	−0.952–0.824
Divergence	0.326	24.41	0.902	67.64	−0.032	−0.157–0.094	−0.960–0.896

The relative test–retest reliability of the different scores for EBPAS showed ICC(A1) (absolute agreement) values >0.705, except for scale 2 (appeal) with an ICC(A1) value of 0.559. The ICC(C,1) (consistency) values were close to the corresponding ICC(A,1) values (Table 3).

The absolute reliability for the total mean score, i.e. the measurement error SEM, was 0.21, and SEM% was about 7%. The SRD was 0.57, and SRD% was about 19% (Table 4); i.e. this represents a real improvement on an individual level. For detailed information about the different mean scores, see Table 2.

**Fig 2:**
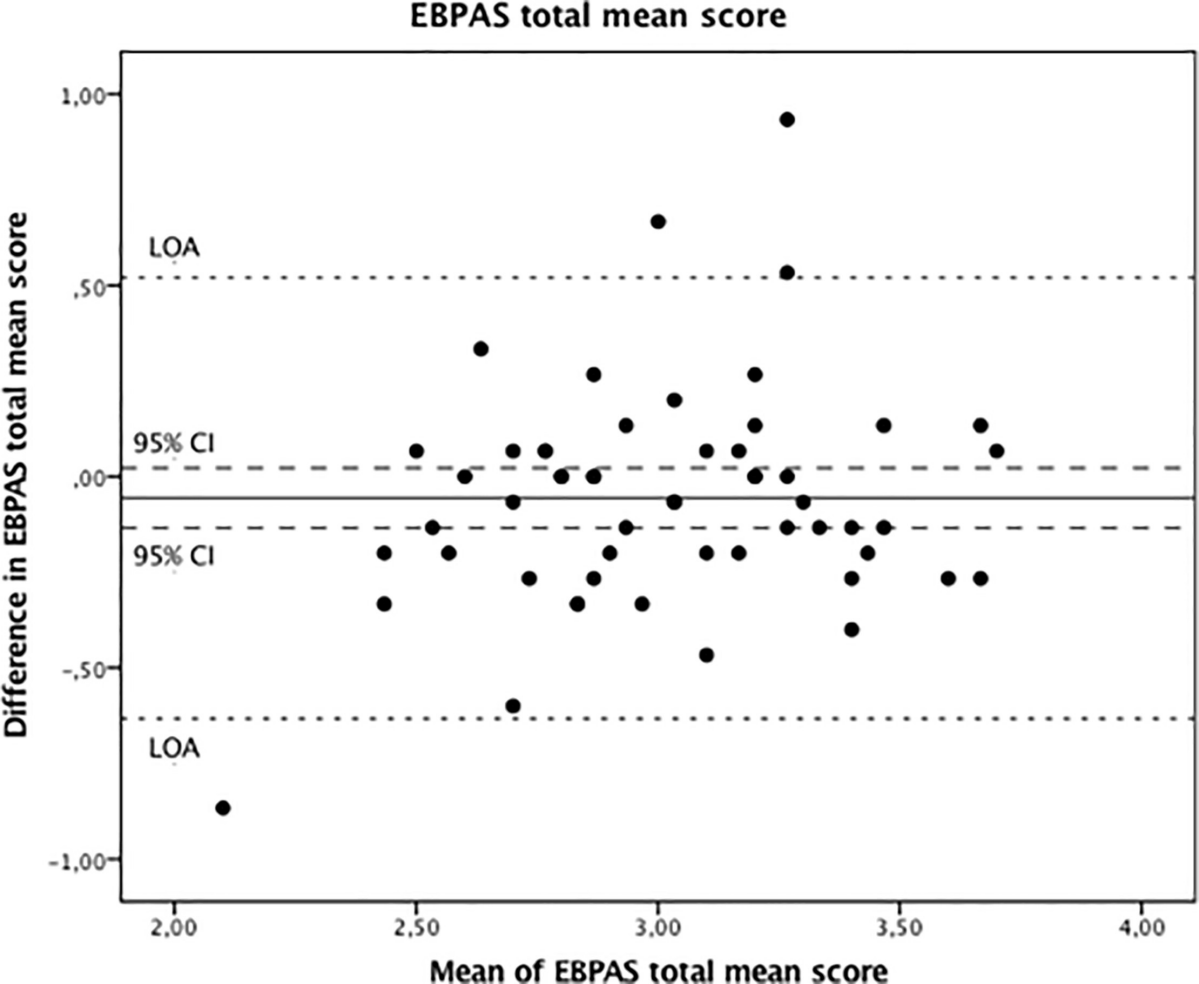
Bland and Altman graphs presenting the Evidence-Based Practice Attitude Scale (EBPAS), total mean score. The difference between the two tests is plotted against the mean of the two tests. Solid line, the mean difference between the two tests; dotted lines, the 95% confidence interval (95% CI) of the mean difference; dashed lines, limits of agreement (LOA).

A Bland-Altman plot of the EBPAS total mean score illustrates that there was no systematic change in the mean (Fig 2). See Table 4 for detailed results of the mean change 95% confidence intervals and limits of agreement for total mean score and each subscale.

## Discussion

The results from this study show that the Swedish version of the EPBAS is a reliable instrument to measure physiotherapist’s attitudes to implementing evidence-based practice. To the authors knowledge, this is the first study presenting values for SEM and SRD of EBPAS, which provides information about measurement error when interpreting clinical relevance.

During the translation process, the interpretation of the translated response options and the term academic researchers were discussed in both the group discussion and by the expert panel. The participants in the group discussion felt that the term academic researchers (item 3) was not applicable for their research experience in their working environment. Research is part of their clinical practice. Research within physiotherapy is a well-established collaboration within clinical practice, which made it difficult to relate to an academic researcher, i.e. only university based. None of the participants considered it to be mandatory according to superiors, business or country. They considered it to be obvious to work with evidence-based practice as registered physiotherapist and a natural component of becoming certified as a physiotherapist.

In the present study, during the translation process, the authors used both an expert committee and back-translation. It is shown in the literature that using an expert committee helps to ensure accurate content in the questionnaire during the translation. Back-translation was considered to have a moderate impact [11]. In a cross-cultural adaptation of the Health Education Impact Questionnaire, an experimental study showed an expert committee, not back-translation, added value. [11] However, in the present study, the authors chose to use back-translation with the intention to show and collect confirmation from the original author of the questionnaire.

EBPAS showed acceptable internal consistency with Cronbach´s alpha coefficients ≥0.74. This is similar to other studies [12–16]. In the present study, the ICC(A.1) and ICC(C.1) values are similar for the mean score and all subscales, see Table 3. This indicates a lack of systematic differences between the first and second tests [6]. Moreover, all F values were consistent with a lack of bias. Subscale 2 (appeal) had lower ICC values than the other subscales. This means that the participants changed their opinion with regard to "appeal" between the first and the second test, however not in a systematic manner (because systematic differences appear to be absent). One might speculate whether the questions concerning "appeal" were vague, making the participants more uncertain. This is a important finding to take into account while using this questionnaire in future studies.

The different mean scores, total and for the four scales, were often slightly higher in the present study in comparison with other studies from Norway, United States, Netherlands and Greece [13–16], which indicates that the sample of physiotherapists in the present study had more favourable and positive attitudes to evidence-based practice in comparison with the study populations from Norway, United States, Netherlands and Greece. This result is also in agreement with the findings from the group discussion during the translation phase, where the physiotherapists expressed that working with evidence-based practice is a natural component in their everyday practice.

To the authors knowledge, this is the first study presenting SRD and SEM for EBPAS. In the present present study the measurement error (SEM) was calculated to 0.21 and the SRD value 0.57, i.e. on a individual level the score has to change more than 0.57 to indicate a clinical relevant change. Both values are important if the instrument is intended to be used to evaluate changes of attitudes to implementing evidence-based practice.

The Evidence-based Practice Attitude Scale (EBPAS) is planned to be used in our future research about implementation of a evicende-based balance training program StayBalance into clinical practice. More specific to assess the implementers attitudes towards new treatments and hopefully give us important knowledge about the implementers acceptability towards the implementation object.

### Study limitations

One limitation in the study might be that the study sample for the reliability assessment was recruited by convenience sampling, i.e. they were therefore self-selected and interested in participation, which may have led to a group of physiotherapist who were already working with and were interested in implementing new evidence-based methods. Furthermore, the gender distribution was skewed, with only a few men (13%) included in the reliability assessment and unfortunately no men in either the expert committee or the group discussion, which make the study population not representative of the physiotherapist population in Sweden.

## Conclusion

The Swedish version of the EBPAS shows mainly good reliability and can be used to assess physiotherapist’s attitudes to implementation of evidence-based practice.

## Supporting information

S1 Dataset (SAV)

S1 Appendix Evidence-Based Practice Attitude Scale. (DOCX)
