# Supplementary material for: Reliability of the Swedish version of the Evidence-Based Practice Attitude Scale assessing physiotherapist’s attitudes to implementation of evidence-based practice
Source: PLoS One. 2019 Nov 25;14(11):e0225467. doi: 10.1371/journal.pone.0225467 (PMC6876878; doi:10.1371/journal.pone.0225467)
Supplement: S1 Appendix — (DOCX) [file pone.0225467.s002.docx]

# Appendix 1
